# Color Vision and Color-Blindness

**Published:** 1896-04

**Authors:** 


COLOR-VISION AND COLOR-BLINDNESS: A Practical Manual for Railroad Surgeons, by J. Ellis Jennings, M. D. (Univ. Penna.), Formerly Clinical Assistant Royal London Ophthalmic Hospital (Moorfields); Lecturer on Ophthalmoscopy and Chief of the Eye Clinic in the Beaumont Hospital Medical College; Ophthalmic and Aural Surgeon to the St. Louis Mullanphy and Methodist Deaconess Hospitals; Consulting Oculist to the Missouri, Kansas & Texas Railway System;

Fellow of the British Laryngological and Rhynological Association; Secretary of the St. Louis Medical Society. Illustrated with one colored full-page plate and twenty-one photo-engravings. Crown octavo, 110 pages. Cloth, $1.00 net. Philadelphia: The F. A. Davis Co., Publishers, 1914 and 1916 Cherry Street.

One of the most serious and remarkable of the many anomalies of the eye is the phenomenon called color-blindness. This consists of an insensibility of the eye to the impression of one or more colors. Sometimes it is only one color; sometimes more than one, and rarely all. The condition of total blindness to all color is so rare that its existence is denied. According to the author of the work under review, "blindness to red or green, or red-green, is the form of the defect most frequently observed."

Those who are blind to red—"red-blindness"—know only two fundamental colors—green and violet. Red appears to such people as "a saturated green of a feeble intensity." Yellow seems to them "a green saturated and intensely luminous."

Green-blind people recognize only two colors—red and violet. Orange appears as a saturated red, but much more luminous. "Yellow is undoubtedly a more intensely luminous red than the spectral red." Blue is an intense violet, violet less intense, but more saturated than normal violet.

In the violet-blind there are two primitive colors, red and green. The blue is a green of moderate luminosity and strongly saturated. This kind of blindness is rare.

Much more detail of these phenomena is given in the book, but the selections here quoted are sufficient to form some general idea of the nature of the defect.

The dangers of color-blindness to human life are obvious to everybody who thinks for a moment of the part that colors play in the running of trains loaded with passengers. To quote from the author: "If we visit one of the great railroad terminal stations at night and view the intricate maze of red, green, blue, and white signals, we at once realize how much depends upon the perfect color sense of the engineer. If he be color-blind, the red and green signals appear to be of the same color and he can only distinguish one as lighter or darker than the other; but a small amount of smoke, fog, snow, and ice destroys this difference and the traveler is at the mercy of his conjecture. That this is a real danger is attested by the many disastrous accidents which have occurred in this country and abroad, and which have been proved to be the result of color-blindness. Notwithstanding all that has been written on this subject, railroad managers still continue to employ men without testing the color-sense. Strange as it may appear, the color-blind employee may perform his duties satisfactorily for many years without causing an accident, and indeed without becoming aware of his chromatic defect."

How then are we to account for this comparative immunity from accident?

The explanation given is as follows: "A locomotive, as we all know, is under the charge of two men—the driver and the fireman. In a staff of one thousand of each, allotted to one thousand locomotives, we should expect, in the absence of any efficient method of examination, to find forty color-blind drivers and forty color-blind firemen. The chances would be one in twenty-five that either the driver or the fireman on any particular engine would be color-blind. They would be one in six hundred and twenty-five that both would be color-blind. These figures appear to show a greater risk of accident than we find realized in actual working, and it is manifest that there are compensations to be taken into account.

"In the first place the term color-blind is in itself in some degree misleading, for it must be remembered that the signals to which the color-blind person is said to be blind are not invisible to him. To the red-blind the red light is a luminous green. To the green-blind the green light is a less luminous red. The danger arises because the apparent differences are not sufficiently characteristic to lead to a certain and prompt identification in all states of illumination and of atmosphere. It must be admitted, therefore, that a color-blind driver may be at work for a long time without mistakes, and it is probable, knowing as he must, that the differences between different signal-lights appear to him to be only trivial that he will exercise extreme caution."

Color-blindness has been found to have its compensations. Thus, one patient says: "I see objects at a greater distance and more distinctly in the dark than any one I recollect to have met with; this I discovered many years before I was aware of my defective error in colors."

A color-blind engraver testifies: "When I look at a picture I see it only in white and black, or light and shade; and any want of harmony in the coloring of a picture is immediately made manifest by a corresponding discord in the arrangement of its light or shade, or as artists term it, the effect. I find at times many of my brother engravers in doubt how to translate certain colors of pictures, which to me are matters of decided certainty and ease."

Another case is related where a physician was asked to test a gentleman connected with a large publishing house, for trouble with colors. The doctor found a condition of red-blindness, and incidentally remarked that the patient would make a good engraver. A member of the firm at once replied "That is just what he is good at, and is our authority on the subject."

In this volume is given a complete *résumé* of the subject and the rules adopted by the Pennsylvania Railroad for the examination of its employees.

